# Agreement and Divergence Between Clinician-Rated and Self-Report Measures of Depression Symptom Severity

**DOI:** 10.1192/j.eurpsy.2026.10587

**Published:** 2026-07-31

**Authors:** L. Rubene-Kesele, E. Dechantsreiter, F. Padberg, D. Bavelier, F. Hummel, N. Lerner, T. Cohen, O. Bonne, Y. Benjamini, M. Nahum, E. Rancāns

**Affiliations:** 1 Riga Stradins University, Riga, Latvia; 2 Department of Psychiatry and Psychotherapy, Ludwig Maximilian University Hospital, Munich, Germany; 3 Campus Biotech & Faculty of Psychology and Educational Sciences, University of Geneva; 4 Neuro-X Institute (INX) and Brain Mind Institute (BMI), École Polytechnique Fédérale de Lausanne, Geneva, Switzerland; 5 School of Occupational Therapy, Faculty of Medicine, The Hebrew University; 6 Department of Psychiatry, Hadassah Medical Center; 7 Department of Statistics and Data Science, The Hebrew University, Jerusalem, Israel

## Abstract

**Introduction:**

Multiple clinician-rated and self-report measures are widely used to assess depression symptom severity. (Ma et al., 2021; Uher et al., 2012) show **moderate to strong correlations**, but also systematic differences. The extent to which these measures can be used interchangeably in clinical practice remains debated.

**Objectives:**

To evaluate the degree of agreement between commonly used clinician-rated (Montgomery–Åsberg Depression Rating Scale [MADRS], Hamilton Depression Rating Scale [HDRS-17]) and self-report (Patient Health Questionnaire-9 [PHQ-9], Beck Depression Inventory-II [BDI-II]) depression symptom severity measures in a clinical sample of patients with major depressive disorder, and to explore whether self-report instruments systematically differ from clinician ratings across severity levels.

**Methods:**

Data was analyzed from 108 patients with major depressive disorder who participated in The DiSCoVeR Trial: ‘Examining the synergistic effects of a cognitive control videogame and a self-administered non-invasive brain stimulation on alleviating depression’. At screening and baseline assesments they completed both clinician-rated scales (MADRS, HDRS-17) and self-report scales (PHQ-9, BDI-II). Pearson correlations were computed among measures, and agreement patterns were visualized with scatterplots.

**Results:**

All measures were significantly correlated (p < .001). Clinician-rated scales correlated moderately with each other (MADRS–HDRS-17: r = 0.59). Self-reported scales showed the strongest association (PHQ-9–BDI-II: r = 0.66). Cross-method correlations were moderate (r = 0.38–0.55), with stronger alignment for MADRS–PHQ-9 (r = 0.55) than for HDRS-17–BDI-II (r = 0.38). Scatterplots revealed variability: patients with identical clinician ratings reported a wide range of self-reported severity. Visual inspection suggested that self-report scores tended to be higher than clinician ratings.

**Conclusions:**

Clinician and self-report depression measures are moderately correlated but not interchangeable. Self-report instruments appear to capture broader variance in symptom perception, while clinician scales show greater consistency with each other. These findings highlight the importance of integrating both perspectives in clinical assessment and research.

**Disclosure of Interest:**

None Declared

